# Application of Freeze-Drying Technology in the Food Industry: A Review

**DOI:** 10.3390/foods15040790

**Published:** 2026-02-23

**Authors:** Angelique Uwineza, Xiaojun Zhang

**Affiliations:** 1 College of Fisheries, Zhejiang Ocean University, Zhoushan 316022, China; inezaange92@gmail.com; 2 Zhejiang Marine Fisheries Research Institute, Zhoushan 316021, China

**Keywords:** lyophilization, food preservation, quality parameters, process optimization, bioactive compounds, sublimation dehydration, rehydration capacity

## Abstract

Freeze-drying, also known as lyophilization, is a state-of-the-art method for preserving food, offering excellent retention properties for nutrients, structure, and taste compared to other drying processes. Freeze-drying yields a product visually similar to fresh produce. However, due to the high energy requirements and operational costs associated with the process, its broader use as an industrial tool is limited. This review encompasses the optimization of all key stages, including pretreatment, freezing, primary drying, secondary drying, and storage. Process efficiency and product quality depend on a variety of factors, including raw material composition, pretreatment strategies (e.g., Pulsed Electric Fields), chamber pressure, shelf temperature, and freezing rate. These parameters are critical control points for determining the final product outcome. Optimizing these parameters is essential; as summarized by recent research, lyophilization effectively protects bioactive compounds, color, flavor, and rehydration ability in various food systems, including fruits, vegetables, meats, seafood, and specialty products. To achieve broader industrial adoption, this gold-standard method requires advancements in process intensification and hybrid drying systems, potentially integrated with intelligent process control. These advances are crucial to enhancing the economic viability of freeze-dried products and maintaining their reputation as the gold standard in creating high-quality, shelf-stable food products. This review consolidates current knowledge into a coherent conceptual model. The model clarifies the deterministic sequence by which adjustable processing conditions direct essential physicochemical changes within the food matrix, thereby defining the product ultimate nutritional, sensory, and stability properties. Establishing this cause-and-effect framework provides a foundation for systematic process improvement and facilitates broader commercial implementation.

## 1. Introduction

Freeze-drying (FD) is a dehydration process that combines freezing and drying. The food is first frozen, and then the pressure is lowered below the triple point to allow for the direct sublimation of ice into vapor [1]. Western European food industries were among the first to adopt freeze-drying technology as an effective dehydration method. This later spread to other nations, becoming one of the most essential drying methods and enabling significant progress in the 21st century food industry [2,3].

The procedure is advanced, effective dehydration technique used to extract moisture from frozen substances, primarily through sublimation [4]. This process removes moisture by transitioning directly from the solid phase to the gas phase and converts ice into vapor under low pressure, entirely avoiding the liquid phase [5]. Freeze-drying preserves the product quality, including its flavor, bioactivities, and other properties. Therefore, it has emerged as an advanced method for extending the shelf life of diverse food products without compromising their original characteristics [6]. Conventional drying methods, such as hot-air drying, offer greater economic efficiency and shorter processing times but often cause thermal degradation of the product, resulting in reduced nutritional value, altered visual characteristics, and impaired rehydration.

In contrast, freeze-drying uses low-temperature sublimation, which more effectively conserves bioactive compounds, sensory quality, and microstructure. Studies on fruits such as pineapple and strawberries demonstrated that freeze-drying enhances ascorbic acid preservation and lead to minimal color deviation (ΔE) compared to other methods [7,8,9]. Despite its high operational costs and long drying times, freeze-drying remains the benchmark method for producing premium, shelf-stable foods. This emphasizes the need to refine freeze-drying protocols, which is the central focus of this review, to facilitate wider industrial implementation [2,10]. This process results in products with a porous structure that enables superior rehydration, minimal shrinkage, and excellent retention of original quality due to low processing temperatures and the absence of oxygen [11,12,13]. However, freeze-dried foods offer extended shelf life without refrigeration and have become an industry standard for high-quality dehydration [12]. While freeze-drying sets the industry standard for producing these foods, its adoption is governed by a critical balance of inherent advantages and significant operational challenges, as summarized in Table 1. The technology was applied to a wide range of foods, including fruits (e.g., strawberries and blueberries), vegetables (e.g., carrots and spinach), meats (e.g., beef and chicken), dairy, instant foods (e.g., coffee and tea), fungi (e.g., mushrooms), and other specialty foods, all of which benefit from the process’s ability to maintain their original form and properties [2,14]. This review aims to clarify freeze-drying mechanisms through a conceptual framework that links key processing variables to their resulting physicochemical changes, and ultimately to the final quality and shelf life of various foods. By structuring the analysis along this causal pathway from parameters to outcomes, a predictive model for process optimization across various food matrices presented.

## 2. Materials and Methods

### 2.1. Literature Search and Study Selection

This review followed a systematic methodology to identify, select, and synthesize the scientific literature on the application and optimization of freeze-drying technology in the food industry. The protocol was designed to ensure transparency, reproducibility, and comprehensive coverage, aligning with established guidelines for systematic reviews and meta-analyses [26,27]. The literature published from 2000 to May 2025, with emphasis on the last decade (2015–2025), was reviewed to capture current advancements. Selected seminal older studies (pre-2000) were included for their foundational knowledge and were sourced from the Web of Science (Core Collection), Scopus, and PubMed.

Google Scholar was used to complement the identification of gray literature, technical reports, and recent preprints. The search strategy utilized a combination of keywords and Boolean operators.

(“freeze-drying” OR “lyophilization” OR “freeze dried”) AND (“food*” OR “fruit*” OR “vegetable*” OR “meat” OR “seafood” OR “dairy”) AND (“quality” OR “optimization” OR “preservation” OR “bioactive” OR “rehydration” OR “sensory”).

Studies were screened against predefined inclusion and exclusion criteria. Inclusion criteria were: (1) original research or review articles in English; (2) a focus on the food applications of freeze-drying; (3) an investigation of process parameters or quality attributes; and (4) publication from 2000 to 2024, with emphasis on the last decade. Exclusion criteria were: (1) exclusive focus on non-food applications; (2) unavailability of full text; (3) conference abstracts without substantial detail; and (4) studies where freeze-drying was not the primary technology. The selection process involved two stages: initial screening of titles and abstracts, followed by a detailed assessment of full-text articles. Data from selected studies were extracted into a standardized template covering food material, process conditions, quality outcomes, and key conclusions [28].

### 2.2. Data Analysis and Synthesis

The extracted data were analyzed thematically to structure the narrative of the review. Findings were synthesized into coherent sections that discussed the freeze-drying process stages; influential parameters; quality characterization methods; applications across food categories; and industrial feasibility, sustainability, and comparative technological analysis. Comparative tables were constructed to summarize data from multiple studies, highlighting trends, optima, and consensuses within the literature. This integrated synthesis approach facilitated the presentation and critical discussion of existing research findings in the subsequent sections. Only statistically significant results were included in the visualization of this review, which to be inserted at the end of Section 2.2.

The synthesized extracted data were organized according to the cause-and-effect logic. The analysis deliberately traced how specific process parameters, such as freezing rate and chamber pressure, governed critical physicochemical transformations, including ice crystal growth and sublimation kinetics. These transformations were directly correlated with measurable product quality attributes, such as rehydration behavior and nutrient retention. This approach provided a structured synthesis that moves beyond descriptive classification, enabling the development of a predictive model for the process.

## 3. Process of Freeze-Drying

**Figure 1:**
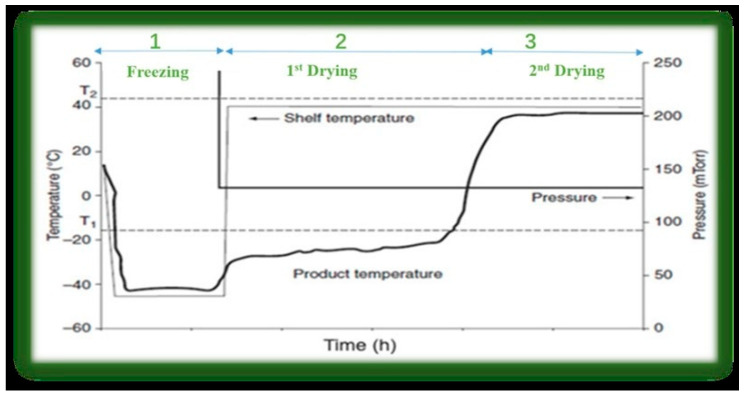
Temperature profile of product during freeze-drying process, where T1 (dotted line) is the collapse temperature and T2 (dotted line) is the glass transition temperature of dry solids, adapted from [35]. under the terms of the CC BY license. Copyright 2020, Bhatta, Janezic, and Ratti.

Garcia-Amezquita et al. found that freeze-drying functions on the principle of sublimation, where water transitions directly from ice to vapor without passing through the liquid phase, particularly at pressures below the triple point (approximately 4.579 mmHg or 611.657 Pa at 0.01 °C) [29]. As outlined by Nowak & Jakubczyk, the process generally consists of three primary stages: freezing, primary drying, and secondary drying, each essential to the quality of the final product [30]. A typical temperature profile is shown in Figure 1. Adequate pretreatment of food before freeze-drying can significantly decrease energy consumption by reducing drying time and improving the quality of the dried products [31]. Based on Baidhe et al., effective post-drying handling is critical to preventing recontamination and preserving quality [32]. As noted by Madelatparvar et al., the initial freezing stage requires careful control of numerous parameters to achieve an appropriate sample temperature [33]. This freezing step is a prerequisite for all materials, whether solid or liquid, before the drying phase [34].

### 3.1. Pretreatment

Pretreatment is a critical preparatory stage in freeze-drying, significantly enhancing drying efficiency and final product quality. These initial steps, including cleaning, sterilization, and reagent addition, aim to standardize raw materials and optimize their performance in subsequent operations [10]. Foods undergo various pretreatments designed to enhance drying efficiency and overall quality. For instance, by altering the microstructure, techniques such as size reduction increase surface area and improve sublimation rates, while blanching inactivates enzymes, maintaining color and nutrients [31,36].

Pulsed Electric Field (PEF) processing electroporates cell membranes, creating micro channels that facilitate water migration and substantially accelerate subsequent drying kinetics. Fauster et al. reported that applying a Pulsed Electric Field (PEF) induces electroporation of cellular membranes in plant tissues, such as apples and potatoes. This structural modification notably decreases undesirable shrinkage and minimizes structural collapse during the sublimation phase. Therefore, the improved porosity enhances the rehydration capacity of the final dried product [37]. Similarly, cold plasma (CP) pretreatment modifies surface properties and inactivates enzymes, thereby improving the retention of heat-sensitive bioactive compounds during lyophilization. For instance, CP-treated green peas exhibit a significantly shorter drying time than the untreated samples, demonstrating a notable improvement in drying efficiency [38]. A study by Li et al. demonstrated that ultrasound-assisted osmotic dehydration (UAD) significantly improved the freeze-drying efficiency and final quality of Sanhua plums.

The pretreatment accelerated moisture removal and better preserved the natural quality of that fruits compared to conventional methods [39]. Wang et al. and Jiang et al. compared three pretreatment methods for strawberry slices: ultra-high pressure (UHP), ultrasound (US), and a combined UHP-US. The result demonstrated that the UHP-US pretreatment was superior. It significantly increased the level of volatile compounds, improved color, enhanced the retention of total phenols and flavonoids, and boosted antioxidant activity, while simultaneously reducing energy consumption compared to untreated samples and other pretreatments [40,41]. These targeted pretreatments overcome major industrial constraints by actively reducing freeze-drying duration, lowering energy demand, and preserving the sensory and nutritional integrity characteristic of high-value freeze-dried products.

### 3.2. Freezing

The freezing process, as outlined by Dalvi-Isfahan et al., consists of three stages: (1) the cooling stage, during which the liquid formulation is cooled from its initial temperature to the freezing point; (2) the phase-change stage, involving the nucleation and growth of ice crystals; and (3) the solidification stage, where ice crystals fully expand until no further growth is possible [42]. Y. Liu et al. demonstrated that freezing is divided into rapid (fast) freezing and slow freezing. Rapid freezing forms small, well-distributed ice crystals that preserve the material’s internal structure, which facilitates adequate rehydration. However, this method leads to a slower sublimation rate during drying. In contrast, slow freezing produces large ice crystals and cracks. These resulting microstructures enhance heat and mass transfer, thereby increasing the drying rate, but they also damage cell membranes and lead to poor rehydration [2]. Dao & Chaeho Moon further visualized this difference, reporting that fast freezing produces numerous small, circular microbubbles, whereas slow freezing results in fewer, larger, millimeter-sized bubbles [43]. According to Obloberdiyev et al., the process involves subjecting food products to extremely low temperatures, typically −40 °C to −80 °C, resulting in the complete solidification of the internal water [6].

#### 3.2.1. Cooling Stage

During the cooling phase, the formulation temperature lowered until the first ice nucleus formed, a point known as the ice nucleation temperature (Tn). Kasper & Friess noted that an aqueous system cooled under atmospheric pressure does not freeze spontaneously at its equilibrium freezing point [44]. For freezing to occur at equilibrium, an ice crystal nucleus must be present at the system freezing point, with no significant temperature gradients.

When these conditions are not met, supercooling occurs: the ability of an aqueous system to remain liquid below its equilibrium freezing point. In this metastable state, water molecules form clusters with relatively long-lived hydrogen bonds, adopting molecular arrangements similar to those of ice crystals [45]. The extent of supercooling significantly impacts the freezing process by the critical nucleation radius and rate. According to L.-P. Wang et al., excessive supercooling, particularly with temperature gradients, can increase nucleation barriers. This can lead to non-uniform nucleation and inconsistent pore size distributions after drying [46]. The supercooling rate also determines its type; Searles et al. identified global supercooling, where the entire liquid volume reaches a similar sub-cooled state and solidification progresses uniformly [47].

#### 3.2.2. Phase-Change Stage

This phase change stage involves the solidification of water within the food product, process influenced by factors such as product size and initial moisture content, which affect cooling and freezing rates [48]. Precise control over crystallization thermodynamics during this stage is critical for determining the final product quality. As Nakagawa emphasizes, achieving the ideal microstructure requires meticulous control that extends beyond merely reaching a target temperature [49]. A key characteristic of this stage is the thermal plateau. Despite the continuous extraction of heat, the product temperature remains nearly constant at its freezing point for a prolonged period. According to Nakaya, the extracted energy is used to break the molecular bonds of liquid water to facilitate ice crystal formation, rather than lowering the temperature [50]. This plateau persists until all freezable water has been transformed into ice.

#### 3.2.3. Solidification Stage

The solidification phase is critical, as it converts the aqueous phase into a stable solid matrix that dictates the final porous structures of the scaffold. This structure, defined by ice dendrite formation, directly influences the efficiency of sublimation during the subsequent drying [51]. The quality of this solid matrix determines key final product characteristics, such as porosity and functionality. Therefore, achieving an optimal structure is paramount for the performance [52]. The primary objective of this phase is to ensure complete conversion of freezable water to ice and to bring the entire product matrix to a temperature that ensures stability under the low-pressure conditions of primary drying. This prevents structural collapse, a phenomenon where the matrix softens and loses porous integrity, leading to process failure and poor-quality products [3].

Freezing rate critically influences ice crystal morphology. According to Jude et al., slow freezing rate promotes larger ice crystals, while faster freezing rate yields smaller ones. The resulting crystal size, in turn, affects the drying rate: larger crystals create larger pores for vapor diffusion, facilitating sublimation and accelerating primary drying [53]. Structural stability during drying is defined by the collapse temperature (Tc). As defined by Roos, Tc marks the specific temperature at which a material can no longer support its own structure, leading to a loss of porous integrity. This threshold is distinct from the glass transition temperature (Tg) [54]. Swarbrick clarifies that Tc is typically several degrees higher than Tg, as the higher matrix viscosity near Tg initially prevents viscous flow and collapse [55]. Overall, the freezing process greatly influences subsequent primary and secondary drying. Key parameters, including cooling rate, degree of supercooling, and nucleation temperature, are critical determinants of final product quality, directly affecting attributes such as residual moisture content and reconstitution time.

### 3.3. Primary Drying (Sublimation)

According to Schoen & Jefferis, primary drying is the phase where ice sublimates from the frozen product. As a critical step, designing an effective process requires the precise establishment of two key parameters: chamber pressure and the intensity of applied heat, with the necessary heat flux being determined by the chosen heating method [56].

Nowak & Lewicki found that setting an appropriate shelf temperature is crucial. When radiation heating is employed, additional factors such as the distance to the material and the infrared spectrum and its intensity must also be optimized [57]. In this stage, ice crystals sublimate directly into vapor under low pressure and temperature, bypassing the liquid phase entirely [58,59]. This removes approximately 90–95% of the water content [2]. During sublimation, the rapid precipitation of dissolved substances can help mitigate the loss of inorganic salts from the food matrix [60]. Babić et al. freeze-dried chicken breast by freezing it at −45 °C for 6 h, followed by primary drying at 0 °C for 8 h and then at 10 °C for 10 h under a constant pressure of 25 Pa [61]. According to Lenaerts et al., Boss et al., and Sasikala, a vacuum is applied to facilitate moisture removal by sublimation, while simultaneously adding heat supplies the necessary latent heat. This energy causes ice to sublimate directly into vapor, which then condenses on cold traps to maintain the vacuum [11,62,63]. This phase is essential for removing the majority of moisture while preserving the structural integrity of the food matrix. Computational modeling of this stage facilitates the prediction of primary drying duration, aiding in thermal optimization and potentially reducing overall energy consumption [64].

Garcia-Amezquita et al. demonstrated that the sublimation front begins at the product surface under reduced pressure and progresses inward as lyophilization continues, enabling the direct transformation of ice to vapor [29]. According to Patel et al. and Tang & Pikal, to initiate and sustain sublimation, vacuum is applied and the shelf temperature is increased to maintain the product temperature typically 2–3 °C below the collapse temperature (Tc) [65,66]. Structural collapse, which occurs if the temperatures exceed Tc, severely degrades product quality by increasing apparent density and residual moisture [67]. This collapse above the glass transition temperature (Tg) adversely affects nutrient retention and rehydration performance, as demonstrated in the tissue of apple, celery, and potato [68].

### 3.4. Secondary Drying (Desorption)

The final stage of the freeze-drying is secondary drying. In this stage, the temperature is increased and the pressure further reduced to efficiently remove the remaining bound water [69]. Li et al. found that at this point, the absence of ice crystals eliminates the risk of melt-back or structural collapse, allowing the use of higher temperatures without damaging the product. Overall, the process removes approximately 98% of the water from the raw material. Freeze-dried products typically exhibit superior freshness and quality upon rehydration compared to those processed by other methods [10]. Waghmare et al. found that secondary drying efficiently reduces residual moisture levels to between 2% and 5% by applying elevated temperature under a sustained vacuum. While these elevated temperatures avoid melt-back, they must be precisely managed to prevent thermal degradation of delicate components [5]. This stage plays a critical role in product stability; by removing bound water, secondary drying significantly enhances the shelf life and quality of the freeze-dried product and prevents its degradation during storage [70].

Researchers have found that the dried porous matrix can absorb water conveyed from the sublimation front through the dried layer. During desorption, the product temperature must remain below a material specific maximum to avoid thermal degradation and an undesirable transition to a rubbery state. For instance, in protein-based products, the temperature should not exceed 40 °C [66,71,72]. The interaction between temperature and material behavior was investigated by Malik et al., who studied gum Arabic solutions (20% to 60% concentrations). Following primary drying at shelf temperatures of 20, 30, and 40 °C, secondary drying was conducted at a constant temperature of 20 °C and 0.1 mbar. The most distinct puffing effect occurred at the 60% concentration when dried at 20 °C and 30 °C, highlighting a significant interaction between composition, temperature, and final structure [73]. Recent studies regarding on modeling approaches for secondary drying employ a dynamic desorption model that functions as a software sensor for process oversight.

This model correlates real-time measurements of the desorption rate with the product residual moisture content and the kinetic parameters of desorption. It enables real-time estimation of (1) the initial water content at the start of secondary drying, (2) the dynamic change in product moisture during this phase, and (3) the time reduced to reach the target final moisture specification [74,75].

### 3.5. Post-Drying Handling

The high hygroscopicity and porosity of freeze-dried products demand immediate and careful post-processing handling to prevent rapid moisture reabsorption and oxidative deterioration, which can compromise structural integrity, nutrient content, and shelf life. Since freeze-drying causes minimal alterations to color and texture, careful post-processing is essential to preserve these qualities and ensure long-term stability [76]. The standard procedure involves breaking the vacuum with an inert gas, such as dry nitrogen, and promptly packaging the product in high-barrier materials, like aluminum foil laminates. This step is often combined with nitrogen flushing or vacuum sealing to actively remove oxygen, creating a protective atmosphere that safeguards product quality [12]. This focus on oxygen exclusion is critical, as residual oxygen (O_2_) is a key determinant of packaged food quality and safety and a primary limiting factor for shelf life [77].

Smiddy et al. and Mohebi & Marquez found that elevated O_2_ levels within packaging, resulting from inadequate barrier materials, flawed sealing processes, improper storage and handling, or physical damage, lead to early spoilage and food deterioration [78,79]. For instance, oxidation alters the taste, texture, and aroma of products while reducing their nutritional value, negatively impacting consumer acceptance and commercial viability [80]. To counteract these issues and extend shelf life, active packaging strategies are employed. These include acidification, vacuum packaging, and modified-atmosphere packaging (MAP), and then the incorporation of functional components such as oxygen scavengers, moisture absorbers, and antimicrobial or antioxidant releasers [81]. Among these, MAP is particularly prevalent and involves replacing air inside a package with a tailored gas mixture (e.g., high nitrogen and low oxygen). This optimized atmosphere effectively maintains product quality and appearance, inhibits microbial proliferation, and prevents recontamination, thereby significantly prolonging shelf life [82].

## 4. Factors Influencing Freeze-Drying Efficiency and Product Quality

The efficiency of the freeze-drying process and the quality of the final product are governed by a set of interdependent parameters. This relationship is best explained by a cause and effect logic: these processing parameters act as primary inputs that drive core physicochemical transformations within the food matrix. These transformations ultimately determine the final product attributes, creating a direct link between operational choices and quality outcomes.

### 4.1. Freezing Conditions

The freezing rate critically determines ice crystal morphology, which governs the extent of structural damage in the food matrix [83]. Proper freezing conditions are essential to preserve the quality of biological materials during freeze-drying. Optimal distribution of unfrozen water, achieved through controlled freezing guided by the frozen-state characteristics, significantly impacts process outcomes such as microorganism survival [84]. Freezing within the lyophilization chamber typically occurs at pressures below 4.58 mmHg and temperatures under 0 °C, conditions that help maintain the integrity of proteins, lipids, and vitamins in sensitive products [85]. W.C and Liu reported that the texture, degree of ripeness, and moisture content in plant tissue substantially influence the freeze-drying process [86]. The effect of freezing rate has been demonstrated across various food matrices. For soursop fruit pulp, Ceballos et al. found that a slower freezing rate of 0.5 °C/min prior to freeze-drying yielded a product with a lower final moisture content, improved solubility, and faster rehydration compared to a fast freezing rate [87].

In apples, a slower cooling rate compromised cell wall integrity, facilitated moisture removal while producing a softer final texture, and enhanced rehydration capacity [88]. Similarly, Ngo et al. reported that freezing rate affected the appearance and aroma of freeze-dried blueberries, with slower freezing resulting in darker color, reduced product volume, and higher volatility of aromatic components [89]. However, the influence of freezing rate is not uniform across all products. Silva-Espinoza et al. found that freezing orange puree at a slow rate (−45 °C in a conventional freezer) versus a fast rate (−38 °C in a blast freezer) did not produce significant differences in the final product’s color attributes or texture [90]. Adequate cooling to a temperature well below the glass transition temperature (Tg) or eutectic point is crucial to ensure complete solidification and prevent structural collapse during primary drying, which is particularly vital for preserving the viability of freeze-dried lactic acid bacteria [91].

### 4.2. Drying Process Parameters

Freeze-drying relies on sublimation, where ice converts directly to vapor under conditions of low pressure and controlled temperature. Essential factors, including the freeze-drying temperature, vacuum pressure, and duration, significantly impact the final product [92]. Managing these parameters requires a careful balance to achieve efficient sublimation and desorption while maintaining the structural integrity and bioactivity of the food matrix. Among the most vital and interconnected factors are the heating conditions and chamber pressure [30]. A critical failure mode described by Ma et al. involves the collapse of the dried layer, which blocks vapor escape. This blockage slows the sublimation rate, reduces heat absorption from the shelf, and causes the frozen core temperature to rise, leading to melt-back and foaming [93]. Dziki et al. demonstrated that raising the plate temperature from 20 °C to 60 °C halved the drying time for kale [94].

Wojdyło et al. found that higher temperatures increase nutrient loss. In strawberries, a higher drying temperature reduced the process but increased vitamin C loss, likely due to oxidation [95]. According to Assegehegn et al., the chamber pressure must be meticulously optimized as it directly controls the driving force for sublimation. A low chamber pressure (Pc) creates a significant pressure differential, allowing efficient sublimation even at lower product temperatures (Tp). Conversely, at high chamber pressures, sublimation only commences if the product temperature (Tp) is raised sufficiently for the ice vapor pressure (Pi) to exceed the chamber pressure (Pc). Sublimation slows or stops entirely if the driving force reaches zero (i.e., ice vapor pressure equals chamber pressure) [45]. For mushrooms, an optimal vacuum of 0.07 mbar was critical; deviations caused structural collapse at lower pressures or extended drying times and vitamin C oxidation at higher pressures [96]. Similarly, the selected pressure range critically influences both the sublimation rate and the efficiency of heat transfer to the product [30].

### 4.3. Material Properties

The physical characteristics of the product load are critical determinants of freeze-drying efficiency. Thinner slices and smaller loads enhance heat and mass transfer, leading to significantly faster drying rates. Waghmare et al. found that the product composition, thickness, and initial solid content all significantly influence process efficiency [5]. Therefore, larger product loads inevitably extend the total process duration, as demonstrated in a study of instant vegetable soup, where increased material load led to longer drying times due to the greater ice mass and complex moisture extraction pathways [30]. Kirmaci et al. demonstrated a linear relationship between freeze-drying duration and sample thickness; 5 mm strawberry slices dried significantly faster (570 min) than 7 mm slices (780 min) [97]. Similarly, higher initial moisture content increases the total mass of water to be removed, directly extending the process duration and energy demand [98]. The material internal composition also presents challenges. High concentrations of soluble solids, such as sugars, increase viscosity and depress the glass transition temperature (Tg). This delays ice formation and lowers the product resistance to collapse during drying [99]. Furthermore, the internal structure of the tissue governs water mobility. Dense or waxy tissues with low cell permeability impede moisture removal, often necessitating physical or chemical pretreatments to facilitate drying [100].

## 5. Characterization of Freeze-Dried Products

### 5.1. Color and Appearance

The color and shape of the freeze-dried products were observed and compared with those of fresh food. Researchers freeze-dried red-fleshed apple slices, which exhibited a modest color improvement compared to fresh apples. When processed under optimized pressure and temperature conditions, the resulting product closely resembled thawed apple, indicating that freeze-drying effectively preserves and even enhances the visual qualities of apples, thereby increasing consumer appeal [101]. Nadal et al. and Sasikala demonstrated that color is quantified through a colorimeter that captures CIE L, a, and b values. The overall color difference (ΔE) is computed to evaluate color preservation, where lower ΔE values signify superior retention of the original color. This method is recognized for maintaining the product original color [63,102].

Comparative studies have shown that freeze-drying minimizes thermal degradation and enzymatic browning. Studies on mushrooms and pineapples have demonstrated that FD yields the lowest ΔE, closely resembling the color of fresh products [103].

$L^{*}$ (degree of light and darkness: 0 is black; 100 is white), $a^{*}$ (degree of red and green: positive value is red; negative value is green), and $b^{*}$ (degree of yellow and blue: a positive value is yellow; a negative value is blue) are quantified using a colorimeter. Based on the three measured data values, the total color difference with the fresh sample can be calculated by

$$∆E=\sqrt{{\left(L^{*}-{L_{0}}^{*}\right)}^{2}+{\left(a^{*}-{a_{0}}^{*}\right)}^{2}+{\left(b^{*}-{b_{0}}^{*}\right)}^{2}}$$

where ${L_{0}}^{*}$, ${a_{0}}^{*}$, and ${b_{0}}^{*}$ are the $L^{*}$, $a^{*}$, and $b^{*}$ of the standards [104]. This property is primarily used for vegetables and fruits.

Researchers have shown that dehydrated and dried pineapple can be processed through various methods, including convection drying, microwave drying, and freeze-drying, among others. As shown in Table 2, the findings indicated that those treated with freeze-drying closely resembled the fresh samples. In contrast, those subjected to the other drying methods exhibited significantly larger values compared to the fresh samples [7].

### 5.2. Nutritional and Functional Properties

Studies have demonstrated that freeze-dried products maintain a greater concentration of proteins, carbohydrates, total sugars, and vitamin C compared to those processed by solar, room-temperature shaded, hot-air, and microwave drying. These samples exhibited elevated levels of total free amino acids and better preservation of bioactive compounds, resulting in enhanced antioxidant activities and confirming their superiority for nutrient retention [70,105].

Based on Ullah et al., freeze-dried products demonstrate an increased protein content up to 30.63, total amino acids of 30.48%, and essential amino acids of 18.92% of the dry weight. Mineral concentrations are generally higher in freeze-dried samples, except for phosphorus and sulphur, further supporting the technique efficacy in preserving proteins, amino acids, and minerals relative to alternative drying methods [106]. According to Naliyadhara & Trujillo, convective drying methods like hot-air drying and atmospheric freeze-drying often reduce antioxidant levels due to prolonged thermal exposure [107].

This is particularly evident in meat processing, where hot drying alters color, aroma, and taste; causes considerable nutrient loss; and results in a product with poor water retention and a tough texture. Freeze-drying, however, better preserves the original nutrients and sensory characteristics of such sensitive foods [93]. According to Li et al., freeze-drying is an advanced technology that effectively maintains the nutritional integrity, sensory qualities, and overall functional properties of food, rendering it a favored method for high-value products [10].

### 5.3. Moisture Content and Water Activity

Waghmare et al. and Maisaroh et al. found that controlling residual moisture is vital for ensuring the stability and extending the shelf life of freeze-dried products, as a lower moisture content inhibits microbial growth and slows detrimental chemical reactions during storage [5,108]. This relationship is best quantified by water activity (a_w_), a critical parameter intrinsically linked to moisture content. A lower a_w_ generally correlates with greater physical and chemical stability, leading to a significantly extended shelf life [109]. Research into hybrid drying methods underscores the importance of process optimization. For shrimp, a hybrid method combining electrohydrodynamic (EHD) drying with freeze-drying (FD) has been investigated. The results demonstrated the clear advantage of the combined technique; compared to FD or EHD used individually, the EHD-FD hybrid process required less total drying time. Furthermore, products from the combined method exhibited significantly reduced shrinkage, a higher rehydration rate, and superior overall sensory quality [110].

Freeze-dried (VFD) products typically achieve very low a_w_ values (often <0.2), enabling stable room-temperature storage without refrigeration. This low a_w_ level prevents enzymatic browning, inactivates enzymes, and retards lipid oxidation. Therefore, understanding and precisely controlling water activity is fundamental to enhancing the safety, stability, and shelf life of freeze-dried foods [111]. The impact of material properties is evident in meat processing. For instance, Aykin & Erbas found that the muscle type significantly (*p* < 0.05) affected the final moisture content of freeze-dried beef. The moisture content of the biceps femoris and semimembranosus muscles decreased from 76.70% to 1.86% and from 76.11% to 2.45%, respectively, yet they reached approximately similar final water activities (0.05 and 0.07) [112].

### 5.4. Rehydration

Rehydration capacity is a critical quality attribute of freeze-dried food, directly reflecting the preservation of structural integrity during processing. A high-quality product should quickly and fully regain its original form upon rehydration, which is a key determinant of consumer acceptability [113]. The exceptionally high specific surface area of freeze-dried products enables rapid and complete water absorption, a property crucial for functionality and user convenience. The rehydration ratio (RR) is defined as the weight of the rehydrated product divided by the weight of the dry product. An increased RR signifies improved water absorption and reduced structural collapse, thereby enhancing the quality [114]. Researchers consistently indicate that freeze-dried products typically outperform those dried by other methods. Freeze-dried tomatoes rehydrate four times faster than hot-air-dried samples and six times faster than infrared-dried samples [115]. The rehydration rate is also influenced by water temperature, with higher temperatures generally accelerating the process [116]. In freeze-dried potatoes, poor rehydration is linked to low porosity. Electrical pretreatment addresses this limitation by improving porosity, which significantly increases the rehydration ratio and enhances the texture and overall quality of the rehydrated product compared to untreated samples [117].

### 5.5. Quality Properties

Hawlader et al. compared the quality of guava powder produced by three drying methods: heat pump drying (relative humidity = 10%, air velocity = 0.7 m/s, temperature = 45 °C, duration = 8 h), vacuum drying (chamber pressure = 15,000 Pa, temperature = 45 °C, duration = 8 h), and freeze-drying (freezing at −20 °C for 24 h, followed by drying at a vacuum pressure of less than 613.2 Pa with a shelf temperature of 10 °C for 24 h). Freeze-dried guava powder yielded a product with better porosity, color, rehydration capacity, and nutrient retention. Notably, it preserved 63% of the original vitamin C compared to only 25% retention by the other methods, making it the most preferred product in the evaluation [8]. Gümüşay et al. investigated the impact of sun-, oven, vacuum oven, and freeze-drying techniques on the levels of phenolics, antioxidant capacity, and ascorbic acid in tomatoes. The freeze-dried tomatoes exhibited approximately double the phenolic content compared to those subjected to other drying methods [118]. This superiority was great, as freeze-drying avoided the high temperatures used in other methods. Elevated temperatures activate oxidative enzymes and cause structural damage that releases peroxidase and hydrolytic enzymes, all of which accelerate the degradation of sensitive phenolic compounds [119].

### 5.6. Physical and Structural Properties

A study by Waghmare et al. found that the freeze-drying process results in a highly porous, rigid structure due to the sublimation of ice, which significantly reduces bulk density and yields a lightweight product. This structural change is essential because it preserves both the nutritional content and key physicochemical properties of the food, such as appearance, aroma, taste, and texture. As a result, freeze-dried products maintain higher quality compared to those processed using other drying methods [5]. The superior rehydration ability of freeze-dried foods is largely attributed to this porosity. However, the same structure leads to pronounced brittleness and mechanical fragility, which directly impact the product handling stability and terminate in a characteristically crispy or crunchy texture, a sensory experience that stands in sharp contrast to the mouthfeel of the original food [30]. Jie Li et al. demonstrated that excessive shrinkage in freeze-drying can occur if the product temperature exceeds its collapse point, compromising the porous structure and impeding rehydration. Proper process control is essential to minimize shrinkage and maintain the quality of the final product [120]. The microstructure and pore morphology of freeze-dried materials are largely determined during the initial freezing phase. When freezing is rapid, it yields a fine, homogeneous, porous network that better preserves cellular integrity. In contrast, slower freezing promotes the formation of larger and more irregular pores, which can compromise the material mechanical strength and lead to undesirable textural or functional flaws in the resulting product [121].

## 6. Impact of Freeze-Drying on Food Quality Attributes

Freeze-drying is particularly effective in preserving high-value and perishable foods. The technology is particularly valuable for high-moisture plant materials. Drying significantly reduces both water content and water activity, which inhibits microbial growth, decreases product weight for easier transport and storage, and extends shelf life, enabling year-round availability [122]. This technology can transform fruits and vegetables into dehydrated products by removing water, while still retaining the chlorophyll, vitamins, and other bioactive nutrients. This technique can be utilized to encapsulate various fruit and vegetable extracts, like whole berries, fruit pieces, and powders. This process expands the spectrum of food applications while maintaining quality [123]. Outcomes across fruits, vegetables, meats, seafood, and specialty products are discussed in this section.

### 6.1. Changes in Fruit Qualities During Freeze-Drying

Freeze-drying preserves 90–95% of the original quality of fruits, significantly surpassing hot-air drying and showing superior nutrients compared to other methods. This results in higher overall product quality [9].

**Figure 2:**
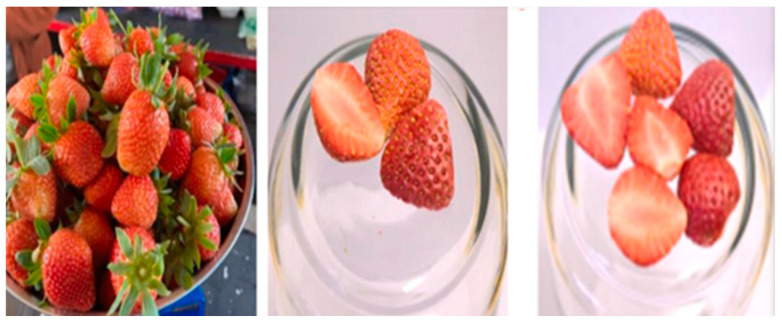
Visual comparison of fresh strawberry, untreated freeze-dried strawberries (FDSs), and osmotic dehydration-pretreated FDSs respectively. The pretreated sample shows superior color retention and texture, aligning with the improved sensory qualities.

Researchers freeze-dried strawberries, resulting in outstanding visual and sensory characteristics and enhancing parameters like lightness, redness, and yellowness, thereby improving their color properties. The total phenolic content (TPC) and antioxidant activity (AA) were only slightly affected by the FD temperature and the methods of pretreatment [124,125]. Fajar Falah et al. showed significant improvement in their previous results. The untreated freeze-dried strawberries (FDSs) retained the natural shape of a strawberry halved vertically, with strong fresh aroma, red color similar to fresh strawberries (Figure 2), robust sour taste, and texture that was neither crunchy nor crispy. In contrast, pretreated FDSs showed improved qualities; they maintained their shape and fresh aroma, but exhibited more vibrant red color, sweeter taste with slight sourness, as well as crunchy and crispy texture [113]. Jude et al. found that freeze-drying is acknowledged to yield superior quality strawberry outcomes compared to conventional drying methods. The expanding market for natural and organic foods further underscores the importance of freeze-drying as a benchmark in the food industry, ensuring minimal processing and the preservation of quality [53].

### 6.2. Changes in Vegetable Qualities During Freeze-Drying

Freeze-drying improves the structural properties of vegetables, notably by reducing shrinkage and enhancing rehydration capacity. This was demonstrated in comparative studies on carrots. Freeze-dried carrots exhibited significantly lower shrinkage (20.83%) compared to hot-air-dried (HAD) samples (35.53%). They also showed impressive rehydration rates and superior retention of aroma and visual appeal [35,126].

Chang et al. highlighted that tomatoes provide a diverse array of antioxidants, encompassing nutritional vitamins (A, C, and E) as well as carotenoids and flavonoids. Freeze-drying effectively retains these components in tomato extracts, making them suitable for use as natural food additives [127]. Lycopene, the principal carotenoid responsible for the red color of tomatoes, is well preserved. Meegahawaththa et al. indicate that tomato peel, rich in lycopene, can be freeze-dried and processed into a powder that maintains both its color and antioxidant properties [128]. Rehydration kinetics of tomatoes are temperature-dependent, with higher temperatures increasing both rehydration capacity and equilibrium water content. Similarly, freeze-drying has been successfully applied to preserve leafy vegetables such as spinach, helping to retain their quality during storage [115,129].

### 6.3. Changes in Meat and Seafood Qualities During Freeze-Drying

Freeze-drying effectively preserves meat and seafood by extending shelf life while maintaining nutritional value and sensory quality. However, the process also causes structural changes, promotes oxidation, and alters the natural color and texture [76]. This technology holds particular importance in major markets such as China, a leading consumer of diverse meats and aquatic products, where research into its application continues to advance [130]. As a result, a wide variety of freeze-dried meat and seafood, including beef, pork, chicken, shrimp, kelp, squid, sea cucumber, and various shellfish, are now commercially available [10].

#### 6.3.1. Meat

S. Lee et al. found that freeze-drying effectively preserves meat products, like beef, poultry, and pork, by reducing deterioration. However, the process induces protein denaturation and oxidation, potentially affecting quality. The extent of these changes depends on freeze-drying conditions, which influence oxidation levels, functional properties, and ultimately the sensory and nutritional quality of the meat [130]. Aykin and Erbas demonstrated that freeze-drying increases the brightness and yellowness of meat, as indicated by significantly higher (*p* < 0.01) L* and b* color values compared to fresh samples [112]. By removing water at low temperatures, freeze-drying alters water component interactions, helping to preserve native protein structure, minimize thermal denaturation, and maintain functional and nutritional properties. Loskota et al. found that the sublimation process, a direct transition from ice to vapor, efficiently removes water while preserving nutrients, making it a valuable technique for enhancing meat quality and shelf life [131].

Meat is an important source of nutrients, including protein, fats, iron, and zinc, which are essential for healthy growth and development. Dal Bosco et al. found that the concentration of these nutrients varies with the type of meat and the processing method used [132]. Furthermore, meat provides essential fatty acids and B-group vitamins, which are vital for nervous system health [133]. However, a substantial portion of the global population lacks access to adequate protein. Expanding the use of freeze-drying technology could help to reduce meat loss and improve the availability of nutritious, shelf-stable meat products [131].

**Figure 3:**
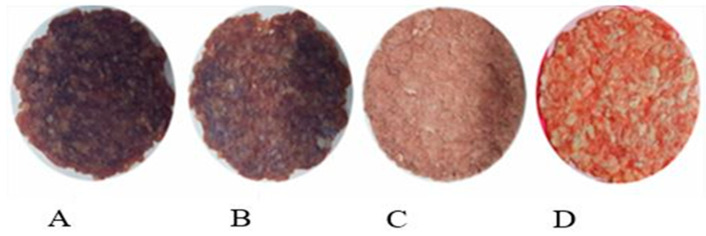
Appearance of meat samples subjected to different drying techniques: (**A**) vacuum drying (VD) at 45 °C, (**B**) ultrasonic vacuum drying (USV) at 45 °C, (**C**) freeze-drying (FD), and (**D**) fresh sample. The superior color preservation in the FD sample is evident. Adapted from [135]. under the terms of the CC BY license. Copyright 2019, Aksoy, A.; Karasu, S.; Akcicek, A.; Kayacan, S.

Rahman et al. compared the vacuum drying (VD) technique at 45 °C to other drying methods and found that VD resulted in the highest ΔE value, indicating significant darkening due to prolonged high-temperature exposure. Ultrasonic vacuum drying (USV) caused less color alteration at 45 °C compared to VD, but freeze-drying (FD) preserved the original color most effectively, as shown in Figure 3 These color changes during drying resulted from oxidation, modifications to the meat surface structure, and non-enzymatic browning reactions, all of which were minimized by the low-temperature freeze-drying process [134].

#### 6.3.2. Sea Foods

Agregán et al. demonstrated that freeze-drying is a method that preserves fish and shellfish, extending shelf life while ensuring high nutritional and organoleptic quality compared to sun-drying and hot-air drying [76]. Wu et al. investigated the effects of FD technology on the quality of aquatic products; sensory evaluations were performed using Antarctic krill as the experimental subject. Following FD treatment, the dried Antarctic krill products demonstrated significant ability to maintain their original protein and fat content; the flavor compounds present in the dried products closely matched those found in the original sample in comparison to HAD (hot-air drying) and vacuum tray drying methods [136]. Hu, Que et al. compared the effects of three drying techniques, specifically HAD, microwave vacuum drying, and vacuum freeze-drying (VFD), on the quality of hairtail fish. The findings consistently indicated the superiority of FD technology. The moisture content of freeze-dried products was low at 4.0%, and they demonstrated enhanced rehydration capabilities compared to products dried using alternative methods [110]. Agregán et al. demonstrated that freeze-drying enhances the preservation of essential and flavor amino acids in scallops. This highlights that modifying process parameters, including heating temperature and material thickness, can elevate rehydration rates to over 83% while simultaneously decreasing energy usage and drying duration by 20% [76].

### 6.4. Changes in Specialty Food Qualities During Freeze-Drying

Coffee and tea are the most popular beverages globally. FD has been used to produce instant tea due to its ability to retain volatile compounds [137]. Kraujalytė et al. found that the instant tea manufactured by FD contained a significant concentration of volatile compounds (318.65 ng/g), which were approximately two to five times greater than the levels found in teas produced using alternative drying methods (68.60 to 143.33 ng/g) [138]. Cheng et al., in the instance of coffee, reported that the concentration of phenolic acids in coffee beans following freeze-drying (FD) increased by 41% compared to fresh green coffee beans [139]. Garlic and ginger have also been reported to be dehydrated using FD. The technique also benefits aromatic vegetables. For garlic, freeze-drying produces a powder with superior color, higher inulin content, and a higher glass transition temperature (44.9–46.2 °C) than forced hot-air drying, which correlates with its low water activity (0.12–0.13). While higher shelf temperatures during freeze-drying can reduce open-pore porosity, the overall apparent porosity increases with drying duration. For ginger, freeze-drying significantly retains gingerols, phenolics, flavonoids, antioxidants, and volatile substances [140,141]. In general, freeze-dried powders are characterized by small particle size, uniform shape, and minimal clumping, making them a reliable method for producing high-performance fruit juice powder [142].

Silva et al. freeze-dried moringa seeds, then peeled and milled them in a domestic blender with varying water volumes of (20, 30, 40, and 50 mL) to form pastes. The pastes were then frozen at −18 °C for 24 h and subsequently dried at −54 °C for 72 h. The resulting powder showed low moisture content, low water activity, and favorable physicochemical properties, as shown in Table 3 [143]. The resulting powders exhibited high luminosity, with L* values exceeding 70. Colorimetric analysis of the a* and b* parameters indicated there was only a statistically significant difference in b* for the natural powder, which displayed predominantly yellow hues. The addition of distilled water during paste formulation decreased chroma (C*), with the 50 mL addition yielding the lowest value. A low browning index in these powders suggested minimal potential for enzymatic or non-enzymatic browning during storage, though powder color can be influenced by multiple variables including genotype, milling, drying method, and storage conditions [144].

Technology is also valuable for dairy products. It is used to dry milk and colostrum, which are rich in bioactive components such as lactoferrin, growth factors, and immunoglobulins. Coşkun et al. compared FD with spray drying for colostrum; it was determined that FD more effectively maintained the levels of protein, IgG, and IgA. Additionally, it is employed in the production of yogurt and cheese products [145].

## 7. Industrial Feasibility, Sustainability, and Comparative Technological Analysis

While freeze-drying delivers superior product quality, its widespread use in industry is limited by high costs and environmental impacts. This section provides a focused analysis of the balance between energy use, expense, and final product quality, and identifies key research gaps that must be filled to advance the technology.

### 7.1. Energy Intensity and Operational Cost Analysis

A primary barrier to the broader adoption of freeze-drying is its substantial energy demand. The process is inherently energy-intensive, requiring both prolonged refrigeration to maintain deep freezing and sustained vacuum pumping to facilitate sublimation. Reported energy consumption ranges from 600 to 1000 kWh per kg of water removed, significantly higher than spray drying or conventional hot-air drying [114,146]. Furthermore, freeze-drying reliance on batch processing significantly increases operating costs, accounting for 50–70% of total production expenses. Compared to continuous manufacturing strategies, this batch-based method creates bottlenecks that limit throughput and present major scalability challenges for high-volume production [147].

### 7.2. Comparative Analysis with Emerging Drying Technologies

To critically evaluate its industrial position, freeze-drying must be compared with emerging and hybrid drying technologies that offer different trade-offs among quality, efficiency, and cost. Although freeze-drying remains superior for preserving heat-sensitive nutrients and microstructure, alternative methods have advanced considerably. For example, microwave-assisted drying enables rapid volumetric heating, significantly shortening process time and reducing energy consumption, but carries risks of uneven heating and localized nutrient degradation [148]. Hybrid systems, such as combined vacuum and hot-air drying, merge the rapid drying rates of conventional techniques with the moderate-temperature advantages of a vacuum. While this improves throughput for certain products, it may compromise the exceptional rehydration capacity and color fidelity characteristics of freeze-drying [149]. Additionally, one notable innovation is air freeze-drying (AFD), which works at near ambient pressure. It presents a feasible solution for certain product categories where a balance between cost and quality is crucial, since it offers an attractive intermediate profile, giving better quality retention than spray drying and greater energy economy than freeze-drying [107]. Thus, the choice of technology hinges on a precise cost–benefit analysis: freeze-drying is the benchmark for premium quality, where high added value justifies the cost, whereas these alternative or hybrid systems present viable, more sustainable options for broader market applications where a slight compromise in quality is acceptable for gains in efficiency and production volume.

### 7.3. Economic and Quality Preservation Trade-Offs

The decision to adopt freeze-drying technology is ultimately guided by a critical balance between maximizing product quality and maintaining economic viability. This balance is strategic, determining the specific market segments where the technique holds a competitive edge. Freeze-drying is economically justifiable primarily for high-value, thermally sensitive ingredients whose superior color, authentic flavor, and high retention of bioactive compounds command a significant market premium.

This includes products such as specialty instant coffees, encapsulated probiotics, premium nutraceutical fruit and vegetable powders, and delicate herbs where preserving the essence of the fresh material is paramount [10,39,139]. For these applications, the higher production cost is often offset by the added market value of a premium-quality ingredient that conventional drying cannot replicate. On the other hand, the significant expense of freeze-drying is hard to justify for bulk commodity products or ingredients where some thermal degradation is sensorily acceptable or nutritionally less important. More affordable techniques like spray drying or convective hot-air drying provide a far more practical solution for the production of many vegetable bulking agents, animal feed components, or shelf-stable staples [114,150]. The choice, therefore, hinges on a detailed cost–benefit analysis, where the marginal quality improvement must clearly outweigh the marginal increase in production expense. Despite the logical nature of this trade-off, quantitative understanding is often lacking in applied research, though many studies convincingly demonstrate the qualitative superiority of freeze-dried products [2,10]. This gap underscores the need for more integrated research approaches. Future work should combine detailed techno-economic modeling incorporating capital investment, energy use, and production throughput with comprehensive sensory and nutritional profiling. Such holistic studies would provide the decision-support frameworks necessary for producers to rationally select the most appropriate drying technology, aligning processing methods with both product goals and market positioning.

### 7.4. Identified Research Gaps and Future Directions

To address these constraints and broaden the industrial applicability of freeze-drying, targeted research must prioritize several interconnected gaps. Promising pathways include the development of optimized hybrid systems, where energy-intensive freeze-drying is preceded by milder dehydration methods like osmotic or microwave drying; integrated approaches can limit total energy use by 30–50% while preserving end product quality. Concurrently, a pronounced scarcity of standardized, cradle-to-gate life cycle assessments (LCAs) hampers informed decision-making; comprehensive analyses comparing the full environmental footprint, including the carbon emissions and water usage of freeze-drying against alternative technologies, are urgently required to guide sustainable process selection. Furthermore, fundamental innovations in process design are essential to overcome the scalability limits of batch operation. Research efforts should be directed toward prototyping continuous freeze-drying systems, integrating enhanced heat recovery mechanisms, and implementing AI-enhanced systems for real-time monitoring and control, which can optimize drying parameters and improve energy efficiency. Successfully advancing these fronts is critical to decouple the exceptional quality attributes of freeze-dried products from their historically high resource intensity, thereby transforming the technology from a premium niche application into a more sustainable and widely viable industrial solution.

## 8. Conclusions

Freeze-drying represents a sophisticated dehydration technology that effectively maintains the structural integrity, sensory quality, and nutritional value of various food products. The success of this method relies on the meticulous control of process parameters, from pretreatment and ice crystal formation during freezing to the precise management of temperature and pressure throughout sublimation and desorption. This review has demonstrated the superior efficacy of freeze-drying over traditional drying techniques in preserving bioactive compounds, color, flavor, and rehydration capacity in fruits, vegetables, meats, seafood, and specialty foods. However, its broader industrial implementation is constrained by high operational costs, substantial energy consumption, and extended processing durations.

As discussed, pathways to mitigate these challenges include the development of optimized pretreatments, hybrid drying systems, and process intensification strategies. Addressing these economic and energetic challenges through such process optimization and technological innovation is crucial for enhancing the viability of freeze-drying. By navigating these trade-offs, freeze-drying can maintain its status as the gold standard for producing high-quality, shelf-stable foods in a competitive global market.

## Figures and Tables

**Table 1:** Advantages and disadvantages of freeze-drying technology.

Aspects	Categories	Details	Reference
Advantages	Preservation of nutritional value and bioactive compounds	Low-temperature sublimation minimizes thermal degradation of heat-sensitive vitamins, polyphenols, and antioxidants.	[2,15]
	Sensory and product quality preservation	Sublimation prevents structural collapse, preserving color, flavor, and aroma and creating a porous matrix for good rehydration.	[6,16]
	Extended shelf life	Reduction in water content and water activity inhibits microbial growth and enzymatic reactions, enabling ambient long-term storage of dried cheese at room temperature.	[6,17]
	Excellent rehydration properties	The highly porous structure formed during sublimation allows for rapid and complete water reabsorption.	[18]
	Lightweight and reduced volume	Removal of most water significantly decreases product weight and bulk, simplifying transport and storage.	[19]
	Versatility in applications	Suitable for a wide range of high-value, heat-sensitive foods (fruits, vegetables, meat, seafood, dairy).	[20]
Disadvantages	High energy consumption and operational costs and prolonged drying time.	The process is energy-intensive due to long cycles of freezing and maintaining a high vacuum, leading to high operational costs.	[1,21]
	Product brittleness and mechanical fragility	The dry, porous structure can be mechanically fragile, leading to potential breakage and texture issues.	[22]
	Packaging requirements	The stringent requirement for specialized, sterile packaging in freeze-drying elevates production costs. Furthermore, the need for specific materials and designs adds a layer of logistical complexity to the packaging process.	[23]
	Limited scalability for large-scale production	The high capital investment required and the reliance on batch-based processing create significant barriers to implementing large-scale continuous production.	[24]
	Potential for quality deterioration during storage	Improper storage above the glass transition temperature (Tg) can lead to loss of texture, color, and flavor over time.	[25]

**Table 2:** Color values of fresh and dried pineapple slices processed by different drying methods [7].

	Color Parameters					
Drying Methods	$L^{*}$	$a^{*}$	$b^{*}$	C	$a^{0}$	∆E
Fresh	69.28 ± 0.45	0.48 ± 0.03	41.86 ± 0.27	41.86 ± 0.27	89.38 ± 0.04	-
Convective drying						
60 °C	68.17 ± 0.49	4.98 ± 0.31	49.56 ± 0.18	49.81 ± 0.17	84.31 ± 0.36	9.09 ± 0.17
70 °C	67.80 ± 0.79	8.16 ± 0.10	48.38 ± 1.74	49.06 ± 1.72	80.46 ± 0.34	10.38 ± 1.13
80 °C	48.70 ± 2.75	13.69 ± 0.58	39.77 ± 2.20	42.06 ± 2.27	71.03 ± 0.30	25.16 ± 2.20
90 °C	40.08 ± 1.29	15.49 ± 0.69	30.34 ± 1.24	34.06 ± 1.42	62.99 ± 0.14	35.30 ± 1.18
Microwave drying						
120 W	65.37 ± 1.50	8.63 ± 0.35	35.62 ± 1.56	36.65 ± 1.53	76.40 ± 0.73	11.35 ± 0.95
350 W	54.63 ± 1.47	11.20 ± 0.58	30.90 ± 1.15	32.88 ± 0.99	70.09 ± 1.41	21.65 ± 0.97
Freeze-drying	74.53 ± 0.60	0.23 ± 0.07	42.95 ± 0.29	42.95 ± 0.29	89.73 ± 0.10	4.83 ± 0.63

**Table 3:** Physicochemical characterization of freeze-dried powder obtained in natura from seeds of moringa.

Physicochemical Parameters	Formation of Freeze-Dried Paste				
	20 mL	30 mL	40 mL	50 mL	CV%
Moisture content (%, d.b *)	1.73 a	1.82 a	1.68 a	1.76 a	3.34
Water activity (a_w_)	0.051 a	0.053 a	0.051 a	0.051 a	1.09
Ashes (%)	2.90 bc	3.02 ab	2.82 c	3.19 a	2.38
Total acidity (%)	0.48 a	0.45 a	0.54 a	0.54 a	3.80
Ph	5.93	6.12	5.40	5.34	2.51
Proteins (%)	33.96 a	33.80 a	34.22 a	34.31 a	1.68
Lipids (%)	27.33 b	27.14 b	38.12 a	30.96 ab	2.05
Carbohydrates (%)	34.06 a	34.20 a	23.14 b	29.77 ab	2.11

Note: The measures followed by the same letter do not differ statistically with each other by the Tukey test to 5% probability. *: Dry basis. CV: Coefficient of Variation.

## Data Availability

No new data were created or analyzed in this study. Data sharing is not applicable to this article.

## Abbreviations

AA	Antioxidant activity
AFD	Air freeze-drying
AI	Artificial intelligence
Aw	Water activity
CIE	Commission Internationale de l’Élaircage (International Commission on Illumination)
CP	Cold plasma
CV%	Coefficient of Variation
d.b.	Dry basis
ΔE	Total color difference
EHD	Electrohydrodynamics
FD	Freeze-drying/Freeze-dried
FDSs	Freeze-dried strawberries
HAD	Hot-air drying
HHP	High hydrostatic pressure
LCA	Life cycle assessment
MAP	Modified-atmosphere packaging
OD	Osmotic dehydration
Pc	Chamber pressure
PEF	Pulsed electric field
RR	Rehydration ratio
Tc	Collapse temperature
Tg	Glass transition temperature
TPC	Total phenolic content
UAD	Ultrasound-assisted osmotic dehydration
UHP	Ultra-high pressure
US	Ultrasound
USV	Ultrasonic vacuum drying
VD	Vacuum drying
VFD	Vacuum freeze-drying

## References

[1] Al Faruq A., Farahnaky A., Dokouhaki M., Khatun H.A., Trujillo F.J., Majzoobi M. (2025). Technological Innovations in Freeze Drying: Enhancing Efficiency, Sustainability, and Food Quality. Food Eng. Rev..

[2] Liu Y., Zhang Z., Hu L. (2022). High efficient freeze-drying technology in food industry. Crit. Rev. Food Sci. Nutr..

[3] Kumar S., Mehra R., Hassani M., Mishra A., Pandey A.K. (2024). Commercial Applications of Freeze Drying in Food Processing. Freeze Drying of Food Products: Fundamentals, Processes and Applications.

[4] Argyropoulos D., Heindl A., Müller J. (2011). Assessment of convection, hot-air combined with microwave-vacuum and freeze-drying methods for mushrooms with regard to product quality. Int. J. Food Sci. Technol..

[5] Waghmare R., Kumar M., Panesar P.S. (2024). Freeze-Drying. Freeze Drying of Food Products: Fundamentals, Processes and Applications.

[6] Obloberdiyev S., Jahongirova M., Tugalova M., Hayitova N., Toshpulatova D. (2025). Technology for Extending the Shelf Life of Food Products Using Freeze-Drying. Am. J. Appl. Sci. Technol..

[7] Izli N., Izli G., Taskin O. (2018). Impact of different drying methods on the drying kinetics, color, total phenolic content and antioxidant capacity of pineapple. CyTA J. Food.

[8] Hawlader M.N.A., Perera C.O., Tian M., Yeo K.L. (2006). Drying of Guava and Papaya: Impact of Different Drying Methods. Dry. Technol..

[9] Liang Y. (2024). Different drying methods’ effects on the nutritional components and flavor of fruits. J. Food Drug Saf. Res..

[10] Li G., Wang Q., Zhou H. (2023). Research on the Application of Vacuum Freeze-drying Technology for Food. E3S Web of Conferences.

[11] Lenaerts S., Van Der Borght M., Callens A., Van Campenhout L. (2018). Suitability of Microwave Drying for Mealworms (Tenebrio Molitor) as Alternative to Freeze Drying: Impact on Nutritional Quality and Colour. Food Chem..

[12] Das T., Nonglait D.L., Rajput D., Arya S.S., Waghmare R.B., Kumar M., Panesar P.S. (2024). Packaging of Freeze-Dried Products. Freeze Drying of Food Products: Fundamentals, Processes and Applications.

[13] Mujumdar A.S., Law C.L., Woo M.W. (2016). Freeze Drying: Effects on Sensory and Nutritional Properties. Encyclopedia of Food and Health.

[14] Berk Z. (2009). Freeze Drying (Lyophilization) and Freeze Concentration. Food Process Engineering and Technology.

[15] Buljeta I., Pichler A., Šimunović J., Kopjar M. (2022). Polysaccharides as Carriers of Polyphenols: Comparison of Freeze-Drying and Spray-Drying as Encapsulation Techniques. Molecules.

[16] Esteller M., Pitombo R., Lannes S. (2005). Effect of freeze-dried gluten addition on texture of hamburger buns. J. Cereal Sci..

[17] Bedir T.B., Kuleaşan H. (2019). Determination of Microbial Properties of Freeze Dried Traditional Cheese. J. Agric..

[18] Khalloufi S., Ratti C. (2006). Quality Deterioration of Freeze-dried Foods as Explained by their Glass Transition Temperature and Internal Structure. J. Food Sci..

[19] Ducklow J., Meidl G. (2022). 399 Freeze Dried pet Food Process. J. Anim. Sci..

[20] Duan X., Zhang M., Mujumdar A.S., Wang R. (2010). Trends in Microwave-Assisted Freeze Drying of Foods. Dry. Technol..

[21] Buana I., Kosasih E.A., Kilic M., Zikri A., Satmoko A., Fauzi M.B. (2025). Enhancing evaporation in vacuum freeze drying: Optimizing airflow in horizontally-connected chambers. Case Stud. Therm. Eng..

[22] Elahi M.F., Wang F., Li Y., Wang L., Yang Y., Yu J., Xu H., Sun B. (2017). Porous Structures from Biobased Synthetic Polymers via Freeze-Drying. Porous Lightweight Composites Reinforced with Fibrous Structures.

[23] Searles J.A., Cherian M. (2015). Alternatives to Vial Lyophilization.

[24] Pisano R., Arsiccio A., Capozzi L.C., Trout B.L. (2019). Achieving continuous manufacturing in lyophilization: Technologies and approaches. Eur. J. Pharm. Biopharm..

[25] Bhandari B., Howes T. (1999). Implication of glass transition for the drying and stability of dried foods. J. Food Eng..

[26] Page M.J., McKenzie J.E., Bossuyt P.M., Boutron I., Hoffmann T.C., Mulrow C.D., Shamseer L., Tetzlaff J.M., Akl E.A., Brennan S.E. (2021). The PRISMA 2020 statement: An updated guideline for reporting systematic reviews. BMJ.

[27] Snyder H. (2019). Literature review as a research methodology: An overview and guidelines. J. Bus. Res..

[28] Liberati A., Altman D.G., Tetzlaff J., Mulrow C., Gøtzsche P.C., Ioannidis J.P., Clarke M., Devereaux P.J., Kleijnen J., Moher D. (2009). The PRISMA Statement for Reporting Systematic Reviews and Meta-Analyses of Studies That Evaluate Health Care Interventions: Explanation and Elaboration. BMJ.

[29] Garcia-Amezquita L.E., Welti-Chanes J., Vergara-Balderas F.T., Bermúdez-Aguirre D. (2016). Freeze-drying: The Basic Process. Encyclopedia of Food and Health.

[30] Nowak D., Jakubczyk E. (2020). The Freeze-Drying of Foods—The Characteristic of the Process Course and the Effect of Its Parameters on the Physical Properties of Food Materials. Foods.

[31] Dziki D. (2020). Recent Trends in Pretreatment of Food before Freeze-Drying. Processes.

[32] Baidhe E., Clementson C.L., Senyah J., Hammed A. (2024). Appraisal of Post-Harvest Drying and Storage Operations in Africa: Perspectives on Enhancing Grain Quality. Agriengineering.

[33] Madelatparvar M., Salami H.M., Abbasi F. (2020). Numerical Study on Parameters Affecting the Structure of Scaffolds Prepared by Freeze-Drying Method. Chem. Chem. Eng..

[34] Liu J., Viverette T., Virgin M., Anderson M., Dalal P. (2005). A Study of the Impact of Freezing on the Lyophilization of a Concentrated Formulation with a High Fill Depth. Pharm. Dev. Technol..

[35] Bhatta S., Janezic T.S., Ratti C. (2020). Freeze-Drying of Plant-Based Foods. Foods.

[36] Sharma S., Barman K., Krishna H., Chaurasia S.N., Mujumdar A.S. (2024). Advances in Freeze Drying to Improve Efficiency and Maintain Quality of Dehydrated Fruit and Vegetable Products. Freeze Drying of Food Products: Fundamentals, Processes and Applications.

[37] Fauster T., Giancaterino M., Pittia P., Jaeger H. (2020). Effect of pulsed electric field pretreatment on shrinkage, rehydration capacity and texture of freeze-dried plant materials. LWT.

[38] Bai J.-W., Li D.-D., Abulaiti R., Wang M., Wu X., Feng Z., Zhu Y., Cai J. (2025). Cold Plasma as a Novel Pretreatment to Improve the Drying Kinetics and Quality of Green Peas. Foods.

[39] Li L., Yu Y., Xu Y., Wu J., Yu Y., Peng J., An K., Zou B., Yang W. (2021). Effect of ultrasound-assisted osmotic dehydration pretreatment on the drying characteristics and quality properties of Sanhua plum (*Prunus salicina* L.). LWT.

[40] Wang C., Zhang L., Qiao Y., Liao L., Shi D., Wang J., Shi L. (2022). Effects of ultrasound and ultra-high pressure pretreatments on volatile and taste compounds of vacuum-freeze dried strawberry slice. LWT.

[41] Jiang D.-L., Wang Q.-H., Huang C., Sutar P.P., Lin Y.-W., Okaiyeto S.A., Lin Z.-F., Wu Y.-T., Ma W.-M., Xiao H.-W. (2024). Effect of various different pretreatment methods on infrared combined hot air impingement drying behavior and physicochemical properties of strawberry slices. Food Chem. X.

[42] Dalvi-Isfahan M., Hamdami N., Xanthakis E., Le-Bail A. (2017). Review on the control of ice nucleation by ultrasound waves, electric and magnetic fields. J. Food Eng..

[43] Dao H.M., Sahakijpijarn S., Chrostowski R., Peng H.-H., Moon C., Xu H., Mangolini F., Do H.H., Cui Z., Williams R.O. (2022). Entrapment of air microbubbles by ice crystals during freezing exacerbates freeze-induced denaturation of proteins. Int. J. Pharm..

[44] Kasper J.C., Friess W. (2011). The freezing step in lyophilization: Physico-chemical fundamentals, freezing methods and consequences on process performance and quality attributes of biopharmaceuticals. Eur. J. Pharm. Biopharm..

[45] Assegehegn G., la Fuente E.B.-D., Franco J.M., Gallegos C. (2020). Freeze-drying: A relevant unit operation in the manufacture of foods, nutritional products, and pharmaceuticals. Adv. Food Nutr. Res..

[46] Wang L.-P., Kong W.-L., Bian P.-X., Wang F.-X., Liu H. (2021). Suppression of ice nucleation in supercooled water under temperature gradients. Chin. Phys. B.

[47] Searles J.A., Carpenter J.F., Randolph T.W. (2001). The ice nucleation temperature determines the primary drying rate of lyophilization for samples frozen on a temperature-controlled shelf. J. Pharm. Sci..

[48] da Silva M.M., de Lima A.B., Gomes J.P., da Silva W.P., de Queiroz R.A., de Lima W.P.B. (2018). The Cooling and Freezing of Parallelepiped-Shaped Solid: Foundations and Application to Food Product. Diffus. Found. Mater. Appl..

[49] Nakagawa K. (2024). Mathematical Modeling of Freeze-Drying Process. Freeze Drying of Food Products: Fundamentals, Processes and Applications.

[50] Nakaya U., Malone T.F. (1951). The Formation of Ice Crystals. Compendium of Meteorology: Prepared Under the Direction of the Committee on the Compendium of Meteorology.

[51] Pawelec K.M., Husmann A., Best S.M., Cameron R.E. (2014). Ice-templated structures for biomedical tissue repair: From physics to final scaffolds. Appl. Phys. Rev..

[52] Chen P.-Y., Chang H.-K., Najman S. (2023). Multifunctional Bio-ceramic Scaffolds and Composites Fabricated by the Freeze Casting Techniques. Bioceramics, Biomimetic and Other Compatible Materials Features for Medical Applications; Engineering Materials.

[53] Jude J., Adu E.A., Maiyanga I.E., Kamaldeen O.S. (2023). Application of Freeze-Drying in Food Processing and Storage. Agric. Res. Environ..

[54] Roos Y.H. (1997). Frozen state transitions in relation to freeze drying. J. Therm. Anal. Calorim..

[55] Fissore D. (2013). Freeze Drying of Pharmaceuticals. Encyclopedia of Pharmaceutical Science and Technology.

[56] Schoen M., Jefferis R. (2000). Simulation of a Controlled Freeze-Drying Process. Int. J. Model. Simul..

[57] Nowak D., Lewicki P.P. (2005). Quality of Infrared Dried Apple Slices. Dry. Technol..

[58] Luo C., Liu Z., Mi S., Li L. (2022). Quantitative investigation on the effects of ice crystal size on freeze-drying: The primary drying step. Dry. Technol..

[59] Jin J., Yurkow E.J., Adler D., Lee T.-C. (2018). Improved freeze drying efficiency by ice nucleation proteins with ice morphology modification. Food Res. Int..

[60] Ling J., Xuan X., Yu N., Cui Y., Shang H., Liao X., Lin X., Yu J., Liu D. (2020). High pressure-assisted vacuum-freeze drying: A novel, efficient way to accelerate moisture migration in shrimp processing. J. Food Sci..

[61] Babić J., Cantalejo M.J., Arroqui C. (2009). The effects of freeze-drying process parameters on Broiler chicken breast meat. LWT.

[62] Boss E.A., Maciel Filho R., de Toledo E.C.V. (2004). Freeze drying process: Real time model and optimization. Chem. Eng. Process..

[63] Sasikala G. (2012). Freeze Drying Plants for the Food and Beverage Industries. IOSR J. Mech. Civ. Eng..

[64] Piechnik E., Stebel M., Palacz M., Haida M., Bodys J., Melka B., Ciesielska A., Smolka J., Nowak A.J. (2024). Simplified computational model of the primary and secondary freeze-drying process of agriculture and marine foods. J. Phys. Conf. Ser..

[65] Patel S.M., Doen T., Pikal M.J. (2010). Determination of End Point of Primary Drying in Freeze-Drying Process Control. AAPS Pharmscitech.

[66] Tang X.C., Pikal M.J. (2004). Design of Freeze-Drying Processes for Pharmaceuticals: Practical Advice. Pharm. Res..

[67] Barresi A.A., Ghio S., Fissore D., Pisano R. (2009). Freeze Drying of Pharmaceutical Excipients Close to Collapse Temperature: Influence of the Process Conditions on Process Time and Product Quality. Dry. Technol..

[68] Karathanos V.T., Anglea S.A., Karel M. (1996). Structural collapse of plant materials during freeze-drying. J. Therm. Anal. Calorim..

[69] Pikal M., Shah S., Roy M., Putman R. (1990). The secondary drying stage of freeze drying: Drying kinetics as a function of temperature and chamber pressure. Int. J. Pharm..

[70] Gat Y., Gawande P. (2024). Freeze-Drying Effect on Nutrients and Their Stability. Freeze Drying of Food Products: Fundamentals, Processes and Applications.

[71] Colandene J.D., Maldonado L.M., Creagh A.T., Vrettos J.S., Goad K.G., Spitznagel T.M. (2007). Lyophilization Cycle Development for a High-Concentration Monoclonal Antibody Formulation Lacking a Crystalline Bulking Agent. J. Pharm. Sci..

[72] Abdul-Fattah A.M., Lechuga-Ballesteros D., Kalonia D.S., Pikal M.J. (2008). The Impact of Drying Method and Formulation on the Physical Properties and Stability of Methionyl Human Growth Hormone in the Amorphous Solid State. J. Pharm. Sci..

[73] Malik N., Muttakin S., Lopez-Quiroga E., Watson N.J., Fryer P.J., Bakalis S., Gouseti O. (2020). Microstructure and reconstitution of freeze-dried gum Arabic at a range of concentrations and primary drying temperatures. Food Hydrocoll..

[74] Pisano R., Fissore D., Barresi A.A. (2012). Quality by Design in the Secondary Drying Step of a Freeze-Drying Process. Dry. Technol..

[75] Fissore D., Pisano R., Barresi A.A. (2011). Monitoring of the Secondary Drying in Freeze-Drying of Pharmaceuticals. J. Pharm. Sci..

[76] Agregán R., Echegaray N., Munekata P.E., Kumar M., Dominguez R., Pateiro M., Lorenzo J.M. (2024). Freeze-Drying of Meat and Seafood Products. Freeze Drying of Food Products: Fundamentals, Processes and Applications.

[77] Banerjee S., Kelly C., Kerry J.P., Papkovsky D.B. (2016). High throughput non-destructive assessment of quality and safety of packaged food products using phosphorescent oxygen sensors. Food Sci. Technol..

[78] Smiddy M., Papkovsky D., Kerry J. (2002). Evaluation of oxygen content in commercial modified atmosphere packs (MAP) of processed cooked meats. Food Res. Int..

[79] Mohebi E., Marquez L. (2015). Intelligent packaging in meat industry: An overview of existing solutions. J. Food Sci. Technol..

[80] Marasca E., Greetham D., Herring S., Fisk I. (2015). Impact of nitrogen flushing and oil choice on the progression of lipid oxidation in unwashed fried sliced potato crisps. Food Chem..

[81] Kelly C.A., Cruz-Romero M., Kerry J.P., Papkovsky D.B. (2018). Stability and Safety Assessment of Phosphorescent Oxygen Sensors for Use in Food Packaging Applications. Chemosensors.

[82] Papkovsky D., Smiddy M., Papkovskaia N., Kerry J. (2006). Nondestructive Measurement of Oxygen in Modified Atmosphere Packaged Hams Using a Phase-Fluorimetric Sensor System. J. Food Sci..

[83] Sabanai R., Suzuki Y., Mizushige T., Uehara N., Inagawa A. (2025). The impact of initial cooling rates on cell preservation in frozen water-dimethyl sulfoxide media: A morphological study. Anal. Sci..

[84] Roos Y.H., Furlong C., Potes N., Roos Y.H., Livney Y.D. (2017). Freezing and Freeze-Drying. Engineering Foods for Bioactives Stability and Delivery.

[85] Nguyen T.D., Chau T.T., Do T.K.L. (2022). Study on Designing and Manufacturing the Freeze Drying System with the Process of Freezing Moist Materials inside the Freeze Drying Chamber to Preserve Valuable Products. J. Tech. Educ. Sci..

[86] Liu W.C., Duan X., Ren G.Y., Liu L.L., Liu Y.H. (2017). Optimization of microwave freeze drying strategy of mushrooms (*Agaricus bisporus*) based on porosity change behavior. Dry. Technol..

[87] Ceballos A.M., Giraldo G.I., Orrego C.E. (2012). Effect of freezing rate on quality parameters of freeze dried soursop fruit pulp. J. Food Eng..

[88] Antal T., Sikolya L., Kerekes B. (2013). Assessment of freezing pre-treatments for the freeze dried of apple slices. Acta Univ. Cibiniensis. Ser. E Food Technol..

[89] Ngo H.T., Tojo S., Ban T., Chosa T. (2017). Effects of Prior Freezing Conditions on the Quality of Blueberries in a Freeze-Drying Process. Trans. ASABE.

[90] Silva-Espinoza M.A., Ayed C., Foster T., Camacho M.d.M., Martínez-Navarrete N. (2019). The Impact of Freeze-Drying Conditions on the Physico-Chemical Properties and Bioactive Compounds of a Freeze-Dried Orange Puree. Foods.

[91] Fonseca F., Girardeau A., Passot S., Wolkers W.F., Oldenhof H. (2021). Freeze-Drying of Lactic Acid Bacteria: A Stepwise Approach for Developing a Freeze-Drying Protocol Based on Physical Properties. Cryopreservation and Freeze-Drying Protocols.

[92] Qian X.-M. (2014). A Method for Optimizing Technical Parameters of the Vacuum Freeze-Drying Process. Kem. Ind..

[93] Ma Y., Wu X., Zhang Q., Giovanni V., Meng X. (2018). Key composition optimization of meat processed protein source by vacuum freeze-drying technology. Saudi J. Biol. Sci..

[94] Dziki D., Polak R., Rudy S., Krzykowski A., Gawlik-Dziki U., Różyło R., Miś A., Combrzyński M. (2018). Simulation of the process kinetics and analysis of physicochemical properties in the freeze drying of kale. Int. Agrophysics.

[95] Wojdyło A., Figiel A., Oszmiański J. (2009). Effect of drying methods with the application of vacuum microwaves on the bioactive compounds, color, and antioxidant activity of strawberry fruits. J. Agric. Food Chem..

[96] Tarafdar A., Shahi N.C., Singh A., Sirohi R. (2017). Optimization of Freeze-Drying Process Parameters for Qualitative Evaluation of Button Mushroom (*Agaricus bisporus*) Using Response Surface Methodology. J. Food Qual..

[97] Kırmacı V., Usta H., Menlik T. (2008). An Experimental Study on Freeze-Drying Behavior of Strawberries. Dry. Technol..

[98] Yao J., Chen W., Fan K. (2023). Novel Efficient Physical Technologies for Enhancing Freeze Drying of Fruits and Vegetables: A Review. Foods.

[99] Roos Y.H. (1998). Phase transitions in frozen water containing sugars and other solutes. J. Pharm. Pharmacol..

[100] Wolkers W.F., Oldenhof H., Wolkers W.F., Oldenhof H. (2021). Principles Underlying Cryopreservation and Freeze-Drying of Cells and Tissues. Cryopreservation and Freeze-Drying Protocols.

[101] Ropelewska E., Lewandowski M. (2024). The Changes in Color and Image Parameters and Sensory Attributes of Freeze-Dried Clones and a Cultivar of Red-Fleshed Apples. Foods.

[102] Nadal M.E., Wyble D., Zarobila C.J. (2014). Color and Appearance. Exp. Methods Phys. Sci..

[103] Martin Michel V.M., Jelen P. (2025). Foods, 2. Food Technology. Ullmann’s Encyclopedia of Industrial Chemistry.

[104] Cinko U.O., Becerir B.B. (2019). Dependence of colour difference formulae on regular changes of colour coordinates in CIELAB colour space. Ind. Textila.

[105] Wei L., Wang W., Hou Y., Wang X., Liu Y., Xie X., Li X., Wang T., Jing B. (2023). Assessment of the Quality Characteristics of Stropharia rugosoannulata Subjected to Five Different Drying Methods. J. Food Process. Preserv..

[106] Ullah R., Akhter M., Khan A.B.S., Yasmin F., Hasan M., Bosu A., Haque M.A., Islam M., Islam A., Mahmud Y. (2023). Comparative Estimation of Nutritionally Important Chemical Constituents of Red Seaweed, Gracilariopsis longissima, Affected by Different Drying Methods. J. Food Qual..

[107] Naliyadhara N., Trujillo F.J. (2025). Advancements in atmospheric freeze-drying: Innovations, technology integration, quality and sustainability implications for food preservation. J. Food Eng..

[108] Hartono L.K., Pongtuluran O.B., Atmaji P., Yuliani S., Setianto W.B., Restiawaty E., Bindar Y. (2025). Freeze- and oven-drying of red ginger juice (Zingiber officinale var. Rubrum): A comparative study on physicochemical properties, bioactive retention, and microstructural characteristics. South Afr. J. Chem. Eng..

[109] Zhao S., Lv J., Li Y., Wang N., Sun G., Zhao Y., Yan Y., Li Q., Tang S., Li Z. (2025). Effects of ultrasonic and high-pressure processing pretreatment on drying characteristics and quality attributes of Angelica keiskei slices prepared by vacuum-freeze drying. LWT.

[110] Hu Y., Huang Q., Bai Y. (2013). Combined electrohydrodynamic (EHD) and vacuum freeze drying of shrimp. J. Phys. Conf. Ser..

[111] Prabhakar K., Mallika E.N., Batt C.A., Tortorello M.L. (2014). Water Activity. Encyclopedia of Food Microbiology.

[112] Aykın E., Erbaş M. (2016). Quality properties and adsorption behavior of freeze-dried beef meat from the Biceps femoris and Semimembranosus muscles. Meat Sci..

[113] Falah M.A.F., Machfoedz M.M., Rahmatika A.M., Putri R.M. (2025). Quality characterization of freeze-dried tropical strawberries pretreated through osmotic dehydration. J. Agric. Food Res..

[114] Ciurzyńska A., Lenart A. (2011). Freeze-Drying—Application in Food Processing and Biotechnology—A Review. Pol. J. Food Nutr. Sci..

[115] Lopez-Quiroga E., Prosapio V., Fryer P., Norton I., Bakalis S. (2019). A model-based study of rehydration kinetics in freeze-dried tomatoes. Energy Procedia.

[116] Lee J.H., Rhim J.W. (2010). Rehydration kinetics of vacuum-dried *Salicornia herbacea*. Food Sci. Biotechnol..

[117] Yaa’ri R., Arazi S., Nussinovitch A. (2024). A Novel Approach to Improving the Rehydration of Freeze-Dried Potatoes through Electrical Treatment. Food Nutr. J..

[118] Gümüşay Ö.A., Borazan A.A., Ercal N., Demirkol O. (2015). Drying effects on the antioxidant properties of tomatoes and ginger. Food Chem..

[119] Toor R.K., Savage G.P. (2006). Effect of semi-drying on the antioxidant components of tomatoes. Food Chem..

[120] Li J., Huang Y., Gao M., Tie J., Wang G. (2024). Shrinkage properties of porous materials during drying: A review. Front. Mater..

[121] Khan S., Khan M.U.A., Ullah Z. (2022). Freeze Drying: A Versatile Technique for Fabrication of Porous Biomaterials. Biomaterial Fabrication Techniques.

[122] Nowacka M., Matys A., Witrowa-Rajchert D. (2024). Innovative Technologies for Improving the Sustainability of the Food Drying Industry. Curr. Food Sci. Technol. Rep..

[123] Kumar Y., Suhag R., Waghmare R.B., Kumar M., Panesar P.S. (2024). Freeze Drying of Fruits and Vegetables. Freeze Drying of Food Products: Fundamentals, Processes and Applications.

[124] Shishehgarha F., Makhlouf J., Ratti C. (2002). Freeze-drying characteristics of strawberries. Dry. Technol..

[125] Biernacka B., Dziki D., Rudy S., Krzykowski A., Polak R., Dziki L. (2022). Influence of Pretreatments and Freeze-Drying Conditions of Strawberries on Drying Kinetics and Physicochemical Properties. Processes.

[126] Rajkumar G., Shanmugam S., Galvâo M.d.S., Neta M.T.S.L., Sandes R.D.D., Mujumdar A.S., Narain N. (2017). Comparative evaluation of physical properties and aroma profile of carrot slices subjected to hot air and freeze drying. Dry. Technol..

[127] Chang C.H., Lin H.Y., Chang C.Y., Liu Y.C. (2006). Comparisons on the antioxidant properties of fresh, freeze-dried and hot-air-dried tomatoes. Food Eng..

[128] Meegahawaththa W.K., Singhalage I.D., Mudannayake D.C. (2020). Tomato (*Lycopersicon esculentum* L.) peel powder as a source of natural antioxidant and a colorant in stirred yoghurt. Food Life.

[129] King V.A.-E., Liu C.-F., Liu Y.-J. (2001). Chlorophyll stability in spinach dehydrated by freeze-drying and controlled low-temperature vacuum dehydration. Food Res. Int..

[130] Lee S., Han S., Jo K., Jung S. (2024). The impacts of freeze-drying-induced stresses on the quality of meat and aquatic products: Mechanisms and potential solutions to acquire high-quality products. Food Chem..

[131] Loskota E., Gramatina I., Kince T. (2023). Application of freeze-drying in meat processing. Food Sci..

[132] Bosco A.D., Mancinelli A.C., Vaudo G., Cavallo M., Castellini C., Mattioli S. (2022). Indexing of Fatty Acids in Poultry Meat for Its Characterization in Healthy Human Nutrition: A Comprehensive Application of the Scientific Literature and New Proposals. Nutrients.

[133] Giromini C., Givens D.I. (2023). Meat in the Diet: Differentiating the Benefits and Risks of Different Types of Meat. Foods.

[134] Rahman M.S., Salman Z., Kadim I.T., Mothershaw A., Hamad Al-Riziqi M., Guizani N., Mahgoub O., Ali A. (2005). Microbial and Physico-Chemical Characteristics of Dried Meat Processed by Different Methods. Int. J. Food Eng..

[135] Aksoy A., Karasu S., Akcicek A., Kayacan S. (2019). Effects of Different Drying Methods on Drying Kinetics, Microstructure, Color, and the Rehydration Ratio of Minced Meat. Foods.

[136] Wu W., Li H., Chen Y., Luo Y., Zeng J., Huang J., Gao T. (2024). Recent Advances in Drying Processing Technologies for Aquatic Products. Processes.

[137] Balentine D.A. (2025). Tea. Kirk-Othmer Encyclopedia of Chemical Technology.

[138] Kraujalytė V., Pelvan E., Alasalvar C. (2016). Volatile compounds and sensory characteristics of various instant teas produced from black tea. Food Chem..

[139] Cheng K., Dong W., Long Y., Zhao J., Hu R., Zhang Y., Zhu K. (2019). Evaluation of the impact of different drying methods on the phenolic compounds, antioxidant activity, and in vitro digestion of green coffee beans. Food Sci. Nutr..

[140] Fante L., Noreña C.P.Z. (2015). Quality of hot air dried and freeze-dried of garlic (*Allium sativum* L.). J. Food Sci. Technol..

[141] An K., Zhao D., Wang Z., Wu J., Xu Y., Xiao G. (2016). Comparison of different drying methods on Chinese ginger (*Zingiber officinale Roscoe*): Changes in volatiles, chemical profile, antioxidant properties, and microstructure. Food Chem..

[142] Taskin O. (2025). Study on the vacuum freeze-drying of banana and impact on powder properties. Case Stud. Therm. Eng..

[143] Silva S.D.N., Almeida F.D.A., Gomes J.P., Barroso A.J.R., Silva P.B., Melo B.A., Silva L.P.F.R., Santos N.C., Matos P., de Moraes M.S. (2019). Moraes, Production and Physical and Physicochemical Characterization Powder in Natura and Freeze-Dried of Moringa Seeds. J. Agric. Sci..

[144] Pathare P.B., Opara U.L., Al-Said F.A.-J. (2013). Colour Measurement and Analysis in Fresh and Processed Foods: A Review. Food Bioprocess Technol..

[145] Coşkun N., Sarıtaş S., Jaouhari Y., Bordiga M., Karav S. (2024). The Impact of Freeze Drying on Bioactivity and Physical Properties of Food Products. Appl. Sci..

[146] Roy S., Rathod G. (2024). Freeze-Drying of Dairy Products. Freeze Drying of Food Products: Fundamentals, Processes and Applications.

[147] Pisano R., Adali M.B., Stratta L. (2022). Modernizing Manufacturing of Parenteral Products. Continuous Pharmaceutical Processing and Process Analytical Technology.

[148] Nwankwo C.S., Okpomor E.O., Dibagar N., Wodecki M., Zwierz W., Figiel A. (2023). Recent Developments in the Hybridization of the Freeze-Drying Technique in Food Dehydration: A Review on Chemical and Sensory Qualities. Foods.

[149] Waghmare R.B., Choudhary P., Moses J., Anandharamakrishnan C., Stapley A.G. (2021). Trends in Approaches to Assist Freeze-Drying of Food: A Cohort Study on Innovations. Food Rev. Int..

[150] Guo X., Hao Q., Qiao X., Li M., Qiu Z., Zheng Z., Zhang B. (2023). An evaluation of different pretreatment methods of hot-air drying of garlic: Drying characteristics, energy consumption and quality properties. LWT.

